# Enhancing the use of technology in the long-term care sector in Canada: Insights from citizen panels and a national stakeholder dialogue

**DOI:** 10.1177/08404704221108466

**Published:** 2022-07-13

**Authors:** Michael G. Wilson, François-Pierre Gauvin, Peter DeMaio, Saif Alam, Anastasia Drakos, Sarah Soueidan, Andrew Costa, Rob Reid, Dorina Simeonov, Andrew Sixsmith, Heidi Sveistrup, John N. Lavis

**Affiliations:** 1 3710 McMaster University, Hamilton, Ontario, Canada.; 2 5543 Trillium Health Partners, Mississauga, Ontario, Canada.; 3 488745 AGE-WELL NCE, Toronto, Ontario, Canada.; 4 1763 Simon Fraser University, Vancouver, British Columbia, Canada.; 5 152971 Bruyère Research Institute, Ottawa, Ontario, Canada.; 6 University of Johannesburg, Johannesburg, South Africa.

## Abstract

Enhancing the use of technology in long-term care has been identified as a key part of broader efforts to strengthen the sector in the wake of the COVID-19 pandemic. To inform such efforts, we convened a series of citizen panels, followed by a national stakeholder dialogue with system leaders focused on reimagining the long-term care sector using technology. Key actions prioritized through the deliberations convened included: developing an innovation roadmap/agenda (including national standards and guidelines); using co-design approaches for the strengthening the long-term care sector and for technological innovation; identifying and coordinating existing innovation projects to support scale and spread; enabling rapid-learning and improvement cycles to support the development, evaluation, and implementation of new technologies; and using funding models that enable the flexibility needed for such rapid-learning cycles.

## Introduction

The COVID-19 pandemic has significantly affected the long-term care sector for many reasons, including residents being at higher risk for serious and life-threating health concerns from COVID-19, exposure to staff with COVID-19, residents living close to each other yet also experiencing significant social isolation, and challenges with providing needed care and support during long periods of lockdown.^1^ The challenges faced during the pandemic were made worse by many long-standing and significant concerns in the sector. These include (but are not limited to) lack of coordination with the sector and between other sectors (eg, primary care, specialty care, rehabilitation, and public health) in health systems, limited collection and use of data to make improvements, limited staff training, satisfaction, and retention, and restrictions in the design and capacity of long-term homes.^2,3^

This has led to many recommendations^4-8^ and initiatives (including the development of standards for long-term care)^9-13^ to strengthen the long-term care sector across the country. Enhancing the use of technology has been identified as a key part of these efforts given its potential to help address some of the biggest challenges in the sector and to improve the health and well-being of residents and caregivers.^3,5,7,14,15^ For example, technology can and has been used to facilitate and enhance communication between facilities, care providers and caregivers, family, and friends (eg, through electronic health records), enhance safety of residents (eg, through location tracking, remote-monitoring technologies, and monitoring use of appliances), and make rooms more accessible (eg, using “Google” or “Alexa” to activate or adjust basic room features such as lights, blinds, temperature, and entertainment).^16,17^ However, many long-term care homes lack adequate internet bandwidth and cyber security and support to enable many of these technologies.^13^

While important, technology needs to be carefully deployed in a way that puts residents, caregivers, and their families at the centre of care. Adopting such approaches can ensure that technology is used to move the needle on achieving the quadruple aim outcomes of enhanced client experiences, improved health outcomes, keeping costs manageable, and enhancing provider experiences. However, focusing the use of technology only as a way to keep costs manageable and ensure efficiency runs the risk of further de-personalizing care and exacerbating many of the existing challenges in the system. For example, technology could be deployed in a way that eliminates tasks for some staff. However, while such a change could free up staff and care providers for more time for interactions and support for residents and caregivers, it could also further reduce opportunities for human interaction if overall staff time is cut.

Addressing complex and pressing system-level policy issues such as these requires the creative interplay of, on the one hand, the best-available data and research evidence and, on the other hand, the tacit knowledge and views and experiences of those who will be involved in or affected by the issue.^18^ Given this, we undertook a multi-component project that involved convening a series of citizen panels, followed by a national stakeholder dialogue with system leaders to identify key actions and priorities from citizens and system leaders to enhance the use of technology in the long-term care sector.

## Approach to convening citizen panels and a national stakeholder dialogue

Our project was conducted in three phases (development of briefing materials, convening citizen panels, and convening the stakeholder dialogue) and guided by an interdisciplinary steering committee that met regularly throughout the project to collaboratively review and revise all project materials and plans for the citizen panels and stakeholder dialogue.

In the first phase, we iteratively developed an evidence brief that synthesized the best-available evidence about key challenges related to enhancing the use of technology in long-term care settings in Canada, three elements of a potentially comprehensive approach to address the problem, and key implementation considerations for the elements. An outline for the evidence brief was developed in collaboration with the steering committee, which was then refined through interviews with 13 key informants that included government policy-makers, organizational leaders, professional leaders, leaders of citizen groups, and researchers from across the country. We used the insights and feedback from the interviews to frame the evidence brief in a policy relevant way and to identify key resources to include. We then refined the evidence brief based on the local data and evidence and relevant systematic reviews (details about the methods are included in the publicly available evidence brief).^2^ We worked collaboratively with our steering committee to turn the evidence brief into a plain-language citizen brief that was used to inform the citizen panels.^19^

In phase two, we convened four virtual citizen panels on January 8, 2021 (with panellists from British Columbia, Alberta, Saskatchewan, and Manitoba), January 11, 2021 (with panellists from Ontario and Quebec), January 14, 2021 (with panellists from New Brunswick, Nova Scotia, Prince Edward Island, and Newfoundland), and on January 15, 2021 in French (with panellists from Ontario, Quebec, and New Brunswick). We recruited panellists in collaboration with AskingCanadians™ panels, which include more than 600,000 Canadians that are affiliated with loyalty programs in Canada and are representative of all the Statistics Canada demographic categories. We sought to engage 14-16 panellists who were diverse in terms of gender, age, sexual orientation, socioeconomic status, ethnocultural background, and geographic residence. We conducted a thematic analysis based primarily on notes taken by the facilitator and secretariat, but recordings of the panels were selectively revisited.

Last, we convened a virtual stakeholder dialogue over two days that was informed by the evidence brief that we pre-circulated and that included a summary of key messages from the citizen panels. Dialogue participants included government policy-makers, organizational leaders, professional leaders, leaders of citizen groups, and researchers that were identified from steering committee members and from suggestions provided by key informants. We aimed to engage system leaders from each province and territory and prioritized those who brought unique views, experiences, and tacit knowledge about the topic *and* who could champion change within their respective constituencies. The dialogue was facilitated by one of us with detailed notes taken by secretariat. Deliberations were thematically analyzed and a summary of key themes was shared with participants and refined at the end of the dialogue. The stakeholder dialogue was convened “off the record” according to the Chatham House Rule and therefore was not recorded.

## Key insights from citizens and system leaders

The citizen panels convened 40 ethnoculturally and socioeconomically diverse citizens over MS Teams from all provinces in Canada. Participants identified as being caregivers, family, or friends of someone currently or recently in a long-term care home. Demographic information of panellists is provided in Table 1. The stakeholder dialogue convened 20 system leaders consisting of eight federal- and provincial-level policy-makers and/or leaders of a health region, two industry representatives, four leaders of stakeholder groups (long-term care, professional, caregiver, and citizen/patient groups and organizations), and six researchers (several of whom were also clinicians). We summarize below the key insights from the panels and the dialogue about the problem, elements of potentially comprehensive approach to address it, and next steps (including implementation considerations).

**Table 1.** Profile of citizen panel participants (n = 40).

Category	N
Age	
18-24	1
25-44	7
45-64	12
65 and older	17
Prefer not to answer	3
Self-identified gender	
Female	17
Male	22
Not identified as male or female	1
Education	
High school	2
Community college	5
Technical school	4
Bachelor’s degree	13
Post-graduate training or professional degree	13
Prefer not to answer	3
Work status	
Self-employed	1
Working full-time	10
Working part-time	3
Unemployed	2
Retired	20
Homemakers	1
Disabled	1
Prefer not to answer	2
Income	
<$20,000	1
$20,000-$40,000	7
$40,000-$60,000	8
$60,000-$80,000	4
>$80,000	11
Preferred not to answer	7
Location	
AB	3
BC	2
MB	3
NB	5
NL	3
NS	2
ON	12
PE	4
QC	5
SK	1
Experience with LTC	
Caregiver of a resident in long-term care	5
Family member of a resident in long-term care	21
Friend of a resident in long-term care	14

### Insights about challenges related to the problem

Panellists identified six important challenges that we describe in Table 2. These include: 1) fundamental issues with long-term care need to be addressed to be able to identify and harness technologies; 2) long-term care homes do not take advantage of technologies; 3) some concerns relying more on technology could reduce human contact; 4) there is a persistent myth that older adults are not interested or able to use technology; 5) the uptake of technologies (if not supported across the system) could further increase inequity in the long-term care sector; and 6) community resources and infrastructure are either not optimally leveraged or are lacking).

**Table 2.** Summary of citizens’ views about challenges related to enhancing the use of technology in long-term care settings in Canada.

Challenge	Description
Fundamental issues with long-term care need to be addressed to be able to identify and harness technologies	Three main challenges related to issues with long-term care that need to be addressed: • Many older adults do not want to end up in long-term care homes, especially after the devastating impact of COVID-19 on residents • Fear of social isolation and loneliness • Limited funding to the long-term care sector resulted in understaffing, overcrowding and poor conditions, lack of recreational activities, infrastructure deficits, and neglecting mental health and social need of residents
Long-term care homes do not take advantage of technologies	• Need to better harness the benefits of technologies in the following areas: ◦ Improving social engagement (eg, communication with caregivers and family outside long-term care homes, as well as social engagement with other residents) ◦ Overcoming impairments (eg, voice-activation technology could be helpful for residents to help overcome the functional impairments that many live with that make using touch-based technology difficult) ◦ Bridging cultural and linguistic barriers between residents and staff ◦ Supporting staff training ◦ Helping caregivers and families access information about residents ◦ Improving transparency and accountability of long-term care homes ◦ Improving resident care and safety • Recurring themes across panels were the need to improve resident care and safety, as well as social engagement
The uptake of technologies (if not supported across the system) could further increase inequity in the long-term care sector	• If uptake and access to technology remains a challenge, health inequities among residents might arise • Disparity in access to technology will persist leading to health inequities without basic standards in place
There are concerns that relying more on technology could reduce human contact	• Technology could be burdensome to staff and require more of their time and resources from long-term care more generally, which could further minimize in-person care and support • There is a need to be careful that technology such as socially assistive robots, tablets, and other devices do not replace human interaction, and that staff does not spend more time supporting the use of technology as opposed to providing direct care • Technologies should instead be adopted in ways that ease certain tasks for staff so that they can provide more direct care, and should also foster greater human interactions with other residents, family members, and the rest of the community
The myth that older adults are not interested in or able to use technology	• Persistent myth that older adults are not interested in or able to use technology was consistently identified, and several panellists insisted that many residents could use technologies with some basic support (particularly those that could help them communicate, break down the cycle of social isolation, and provide entertainment) • Many technologies have not been developed to meet the specific needs of residents (particularly those with physical and cognitive impairments) and long-term care staff was raised as being important to address
Community resources and infrastructure are either not optimally leveraged or are lacking	• Community supports are often not leveraged to fill the gaps in long-term care homes • Lack of affordable high-speed internet access in some areas across the country is a key upstream barrier • Lack of wifi being available for all residents is an issues and, when it is available, it sometimes used only for administrative purposes

In addition to the challenges identified by citizens, dialogue participants highlighted four additional challenges:

1) lack of a comprehensive innovation agenda for long-term care;
2) residents, families, and caregivers are rarely and inconsistently prioritized and meaningfully engaged in technology development and in efforts to strengthen long-term care more generally;
3) technologies are often not attuned to the individual needs of residents and local realities; and
4) the long-term care sector is not an innovative space and the value of technology is often questioned.

### Insights about elements of a potentially comprehensive approach to address the problem

Citizen panellists and dialogue participants deliberated about three elements of a potentially comprehensive approach to address the problem focusing on: 1) ensuring that long-term care homes operate in a context that can support the adoption of appropriate technologies; 2) engaging long-term care home operators, staff, residents, and their caregivers in developing and adopting technologies; and 3) enabling rapid-learning and improvement cycles (ie, making small, yet rapid changes over time) to support the development, evaluation, and implementation of new technologies. The elements were presented to citizens and dialogue participants with a summary of key findings from systematic reviews.^2^ Table 3 provides a detailed summary of values and preferences from citizens and insights from system leaders about the elements.

**Table 3.** Insights from citizens and dialogue participants about three elements of a potential approach to enhancing the use of technology in long-term care settings in Canada.

Element	Possible components of the elements (as described in the citizen and evidence briefs)^2,19^	Key insights from citizen and system leaders
Element 1: Ensure that long-term care homes have the supports they need to use technologies	• Efforts to upgrade existing buildings • Ensure future buildings are designed and built in a way that is appropriate for enabling the adoption of technologies • Ensure community supports for technology use are available (eg, availability of affordable broadband internet connections)	Citizens’ values • Holistic approaches to care and support and the use of technology • Collaboration between long-term care homes and community-based organizations • Excellent care experience (resident, family, and community-centred) • Basing decisions on citizens’ values and preferences Citizens’ preferences • Use co-design approaches to plan the renovation of existing long-term care and the building of new ones, as well as determining priorities (eg, through community advisory committees) • Consider the physical/technological environment, social, cultural, and policy contexts that can support the adoption of appropriate technologies • Ensure collaboration between long-term care homes and community-based organizations (eg, schools, public libraries, and other non-governmental organizations) to get support for the adoption of technologies • Provide access to community supports that should internet access, as well as social programming that could be delivered on-line to support social engagement (eg, leveraging on-line programming of public libraries), engaging volunteers (eg, high-school students) to help teach residents to use technology, and adopting a device-sharing program • Commit to ongoing and meaningful engagement in determining what's needed for upgrading existing buildings, requirements for new buildings, and community supports (eg, design practices that ensure person-centredness in everything, not just use of technology) • Implement advisory boards that are comprised of residents, caregivers, and families (ie, people with lived experience) to inform and support decisions • Improve the care experience of residents by grounding solutions in the values and preferences of citizens Insights from system leaders • This element generated less discussion, but participants agreed with the values and preferences outlined above, and emphasized the need for meaningful and consistent engagement of the type emphasized in element 2
Element 2: Engage long-term care home operators, staff, residents, their caregivers, and the industry in developing and adopting technologies	• Requirements for co-design processes with residents, their caregivers and long-term care operators to develop technologies that: ◦ Meet the needs of residents and caregivers (eg, for communication with caregivers and with clinicians, and keeping residents safe) ◦ Support the operation of long-term care homes (eg, providing training for staff) ◦ Integrate with the broader system (eg, integrated electronic health records, and remote monitoring)	Citizens’ values • Innovation • Excellent care experience (resident, family, and community-centred) • Collaboration between long-term care homes and key stakeholders • Adaptability • Value for money (resource stewardship) Citizens’ preferences for operationalizing values • Use co-design approaches to: ◦ Improve the experiences of residents, caregivers, and family members ◦ Enhance the usefulness and adaptability of technologies for the needs of long-term care residents, caregivers, and families, and ultimately increase use of new technologies ◦ Support greater collaboration among all those involved (long-term care staff and operators, as well as residents, caregivers, and families) Insights from system leaders • Agreed about the need to involving staff, residents, and their caregivers and families in co-designing technologies empowers them to use them more effectively • Consensus that co-design should not only be used for developing technologies, but also a process to re-imagine long-term care through ongoing deliberation with long-term care owners, operators, and staff, the tech industry, as well as residents, families, and caregivers
Element 3- Make small yet rapid changes that are centred on residents, caregivers, and families to support the development, evaluation, and implementation of new technologies	• Engaging in rapid-learning and improvement cycles that are: ◦ Centred on residents and caregivers (eg, by building acceptance for using technology among residents and their caregivers) ◦ Data and evidence driven (eg, by creating centralized platforms to share data and evidence about technologies that can be adopted in long-term care, and insights about their use that can be used to drive learning and improvement cycles) ◦ Supported through aligned system arrangements (eg, by changing governance, financial and delivery arrangements that currently prevent the adoption, evaluation, and continuing modifications of the use of new innovations) ◦ Enabled by supportive competencies and culture (eg, through a long-term care learning collaborative)	Citizens’ values • Standardization • Accountability • Fairness • Innovation • Leadership Citizens’ preferences • Develop and implement national standards and guidelines to enhance long-term care and for associated areas that would have an impact on the use of technology in provinces/territories • Prioritize innovation in long-term care, by ensuring necessary conditions for long-term care homes to innovate on their own and/or implementing a coordinating body and information-sharing platform for sharing innovative solutions • Ensure centralized leadership within each long-term care home and at the regional/provincial level to support scaling up and spreading technological innovations, and to monitor improvements Insights from system leaders • Need to adopt a rapid-learning approach to support the development, evaluation, and implementation of new technologies in long-term care homes in Canada • Enhance standards in existing long-term care homes, which may require addressing challenges from public/private ownership models of long-term care Canada

Overall, citizens highlighted a need for establishing national standards and guidelines for improving long-term care in provinces. Citizens also emphasized the need for co-design approaches to be used in planning the renovation of existing long-term care homes and for building of new ones, and the context of long-term care homes should encompass the broader social, cultural, and policy environments that can support the adoption of suitable technologies. Innovation was identified as a recurrent theme, but some panellists emphasized the need for incremental innovations, while others advocated for radical innovations, which was also deliberated by system leaders in the stakeholder dialogue.

Building on this, the deliberations among system leaders culminated in a consensus about the need for an innovation agenda that could be advanced by focusing on the elements related to engaging long-term care home operators, staff, residents, and their caregivers in developing and adopting technologies, and to enabling rapid-learning and improvement cycles to support the development, evaluation, and implementation of new technologies. However, views on the form and scope of an innovation agenda that should be implemented differed, which we describe below in the section about implementation considerations.

For the element focused on engagement, participants underscored the need to focus an innovation agenda with solutions for what the long-term care sector *should* be. Overall, the participants highlighted this would first require a re-imagining of the sector and a co-design approach. The importance of a co-design approach (which was also emphasized by panel participants) was seen as critical given that insights from those engaged at the frontline (ie, staff, residents, caregivers, and families) in designing technology are important for it to be used more consistently and effectively. Moreover, co-design was viewed as essential for broader efforts to re-imagine long-term care.

Building on this, deliberations about rapid-learning and improvement cycles centred on how to use this type of approach to begin a re-imagining process and build an innovation agenda to address the many large and long-standing issues in the long-term care sector. The key issues emphasized included implementing country-wide standards, enhancing coordination for technology innovation within and between governments, addressing the challenges posed from having varied long-term care ownership models, and ensuring ongoing engagement as described above. Co-design was again seen as fundamental in this type of process to ensure that a rapid-learning approach to enhance technology use in long-term care (and re-imagining of the sector) is grounded on the values, needs and preferences of older adults and the best-available evidence.

### Implementation considerations

While system leaders agreed on the need for an innovation agenda for enhancing the use of technology in long-term care and strengthening the sector, they highlighted three different implementation approaches: incremental, disruptive, and radical innovation. Incremental innovation focuses on setting and implementing short- to medium-term goals for enhancing quality of care and care processes. This was viewed as being achievable within the existing structure of long-term care in Canada. Examples included efforts to establish better communication and data sharing between sectors (eg, with the primary, specialty, rehabilitation, and public-health sectors), using technology for routine care and for leisure (eg, to address isolation among residents). Doing so would require addressing barriers to technology adoption, including those with procurement processes, lack of financial incentives and/or flexibility to do things differently and continuing to work through long-standing issues with data confidentiality, privacy and ownership. In contrast, disruptive innovation was seen as an opportunity for technology to be used as part of efforts to significantly change the long-term care sector. For example, some suggested moving from a more institutionalized setting to one that is more home like and therefore person centred. Last, adopting a radical innovation agenda was viewed as an opportunity to re-think long-term care for the digital age. Many system leaders who participated in the dialogue supported this option as they viewed the combination of rapidly emerging digital solutions with increasingly tech-focused residents, families and caregivers as an opportunity to re-engineer the sector. Moreover, it was noted that this approach might be needed to support an ageing baby boomer generation that seems to have little interest in entering long-term care in its current form.

## Next steps

System leaders highlighted that the sustained interest in the need to reform the long-term care sector during the COVID-19 pandemic and in recovery stages can be capitalized on to pursue fundamental changes to the sector. With this window of opportunity, system leaders identified several inter-connected next steps to advance an innovation agenda for long-term care, including: 1) using a co-design approach in all efforts to reform the long-term care sector; 2) identifying and coordinating existing innovation projects to support scale and spread, and develop an innovation roadmap/agenda; 3) using technologies in a way that enables person-centred care and support in long-term care; 4) modifying policy and organizational processes in the sector to use decision-making processes that foster and implement innovation over time by prioritizing making small yet rapid changes that are centred on residents, caregivers, and families; and 5) using funding models that enable the flexibility needed for such rapid-learning cycles. Collectively, these actions were viewed as the foundation upon which a re-imagined long-term care sector could be built. Indeed, in the time since the citizen panels and the stakeholder dialogue were convened in early 2021, there have been national efforts to create standards in long-term care^12,13^ and to establish priorities for reforms in some provinces.^20-22^ Our findings will be important for operationalizing such standards and priorities.
